# Profitable Tree Planting

**Published:** 1874-12

**Authors:** 


PROFITABLE TREE PLANTING. — It has been feared that Peruvian bark, from which the various preparations of quinine are made, would become so nearly extinct as to be no longer pro- [illegible]

since, the cultivation of the cinchona was taken up by the British Government in India. As one of the results of this planting, the *Chemist and Druggist* says that, at a recent sale in Mincing Lane, a lot of mossed crown bark (*Cinchona efficinalis*), from the Nilgheri plantation, fetched the prodigious price of 5s. 9d. a pound. Altogether, 23,645 pounds of Nilgheri cinchona bark was sold on this occasion, and the total sum realized was £3,350, the average price fetched being about 2s. 10d. a pound—a very high average. The total cost of the introduction of the cinchona into India, including the cost of Mr. Markham's expedition to Peru and India, has from first to last been £70,000, and the annual sales now realize a net profit of from £4,000 to £5,000 a year. The experiment suggested by Sir George Clark, and carried out by Mr. Markham, has therefore proved an assured and splendid success.

